# From circRNAs to fusion circRNAs in hematological malignancies

**DOI:** 10.1172/jci.insight.151513

**Published:** 2021-11-08

**Authors:** Loelia Babin, Elissa Andraos, Steffen Fuchs, Stéphane Pyronnet, Erika Brunet, Fabienne Meggetto

**Affiliations:** 1 CRCT INSERM, UMR1037, Toulouse, France.; 2 Toulouse III University-Paul Sabatier, UMR1037 INSERM, UMR5071 CNRS, Toulouse, France.; 3 The Toulouse Cancer Laboratory of Excellence (TOUCAN), Toulouse, France.; 4 Department of Pediatric Oncology, Charité University Berlin, Berlin, Germany.; 5 Berlin Institute of Health (BIH), Berlin, Germany.; 6 German Cancer Consortium (DKTK), Partner Site Berlin, German Cancer Research Center (DKFZ), Heidelberg, Germany.; 7 Imagine Institute INSERM Joint Research Unit 1163, Laboratory of Genome Dynamics in the Immune System, Paris, France.; 8 Paris Descartes-Sorbonne University, Imagine Institute, Paris, France.

## Abstract

Circular RNAs (circRNAs) represent a type of endogenous noncoding RNA generated by back-splicing events. Unlike the majority of RNAs, circRNAs are covalently closed, without a 5**′** end or a 3**′** poly(A) tail. A few circRNAs can be associated with polysomes, suggesting a protein-coding potential. CircRNAs are not degraded by RNA exonucleases or ribonuclease R and are enriched in exosomes. Recent developments in experimental methods coupled with evolving bioinformatic approaches have accelerated functional investigation of circRNAs, which exhibit a stable structure, a long half-life, and tumor specificity and can be extracted from body fluids and used as potential biological markers for tumors. Moreover, circRNAs may regulate the occurrence and development of cancers and contribute to drug resistance through a variety of molecular mechanisms. Despite the identification of a growing number of circRNAs, their effects in hematological cancers remain largely unknown. Recent studies indicate that circRNAs could also originate from fusion genes (fusion circRNAs, f-circRNAs) next to chromosomal translocations, which are considered the primary cause of various cancers, notably hematological malignancies. This Review will focus on circRNAs and f-circRNAs in hematological cancers.

## Introduction

Only 2% of the human genome encodes proteins. The rest can be transcribed into noncoding RNAs (ncRNAs) (1), which are divided into two categories: 1) housekeeping ncRNAs (ribosomal [rRNA], transfer [tRNA], small nuclear [snRNA], and small nucleolar [snoRNA]) and 2) regulatory ncRNAs, which can be classified as small ncRNAs (<200 nucleotides), such as microRNA (miRNA), and long ncRNAs (lncRNAs) (>200 nucleotides) (2, 3). Circular RNAs (circRNAs) are a class of noncapped, nonpolyadenylated lncRNAs characterized by a covalently closed loop structure and as such are extremely stable. CircRNAs were initially considered background noise due to splicing errors; however, high-throughput sequencing technologies and bioinformatic algorithms reveal that circRNAs are abundant, have important biological functions, and regulate critical pathways in malignant pathologies. Thus, circRNAs have potential as therapeutic targets or as powerful biomarkers (4).

## CircRNA biogenesis and degradation

CircRNA length varies between hundreds and thousands of nucleotides. As the majority of the identified circRNAs are composed of RNA polymerase II–transcribed exons of either pre-mRNAs (encoding well-known proteins) or pre-lncRNAs, the constitutive splicing machinery is believed to be involved in their biogenesis.

CircRNA biogenesis involves a back-splicing event, whereby a downstream 5′ splice (GU donor site) is joined to an upstream 3′ splice (AG acceptor site) (5). CircRNAs may originate from either coding or untranslated exons (exonic circRNAs, EcircRNAs), introns (circular intronic RNAs, or ciRNAs; ref. 6), or exon-intron combinations (EIcircRNAs). tRNA intronic circRNAs are formed by splicing a pre-tRNA intron (7–9) and much less frequently from intergenic regions or antisense RNAs (10). Currently, most of the identified circRNAs are EcircRNAs.

**Figure 1:**
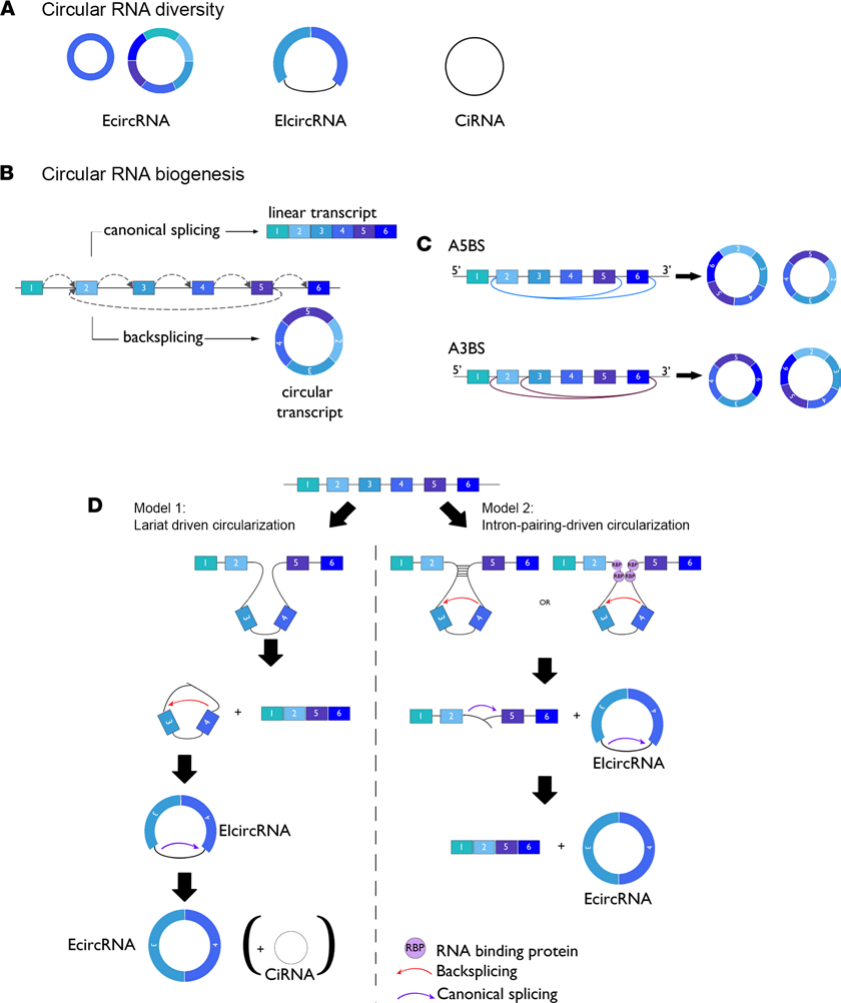
CircRNA diversity and biogenesis. (**A**) Pre-mRNA can be processed through either canonical splicing to produce linear RNAs or back-splicing to generate circRNAs. CircRNAs are mainly divided into three categories based on their components: exonic circRNAs (EcircRNAs) are exclusively composed of exons and represent the largest group of circRNAs, intronic circRNAs (ciRNAs) are exclusively composed of introns, and exon-intron circRNAs (EIcircRNAs) are composed of exon-intron sequences. (**B**) A single gene locus can produce both linear and circular transcripts by canonical splicing or back-splicing mechanisms, respectively. (**C**) A single gene locus can produce multiple circRNAs that share the same back-splice site through a mechanism called alternative back-splicing (ABS), due to the competition among inverted complementary sequences across introns that bracket the circRNA-forming exons. There are two types of ABS events, alternative 5′ back-splicing (A5BS) and alternative 3′ back-splicing (A3BS). In an A5BS event, two or more downstream 5′ back-splice sites are alternatively joined with the same upstream 3′ back-splice site, while for A3BS circRNAs, the same downstream 5′ back-splice site is alternatively joined with two or more upstream 3′ back-splice sites. (**D**) Two main models have been proposed for circular RNA formation. Model 1: Lariat-driven circularization (exon skipping). Splice donor in the 3′ end of exon 2 covalently splices to the splice acceptor in the 5′ end of exon 3 and forms a lariat via exon skipping. Model 2: Intron-pairing-driven circularization. Intron 2 and intron 4 form a circular structure via base-pairing (-) or trans factors such as RNA-binding proteins (RBPs). The introns are then removed and EIcircRNA, EcircRNA, and intron lariat (ciRNA) are formed.

A process called alternative back-splicing (ABS) allows several circRNAs to be generated from a single gene locus. Two ABS processes have been described: 1) alternative 5′ back-splicing (A5BS) in which one 3′ acceptor site indifferently fuses to various downstream 5′ donor sites and 2) alternative 3′ back-splicing (A3BS) in which one 5′ donor site indifferently fuses to different upstream 3′ acceptor sites (11) (Figure 1).

Single- or multi-exon forms represent up to 80% of all circRNAs. During conventional pre-mRNA splicing, an exon-skipping event may occur concomitantly, promoting the formation of EcircRNA, circRNA, and mRNA splice variants. This process is known as lariat-driven circularization. In some cases, and following the back-splicing process, introns are retained in the circRNAs, generating EIcircRNAs. A recent study has identified thousands of circRNAs generated from intronic sequences through back-splicing processes (Figure 1) (12).

CircRNA generation is also promoted by trans factor–facilitated back-splicing events that bring distant 5′ and 3′ back-splice sites into close proximity. This process is known as intron-pairing-driven circularization (7). Among these promoting factors, intronic complementary sequence pairing (GU-rich and C-rich elements) as well as various RBPs are implicated. RBPs bind double- or single-stranded RNA and are crucial for all aspects of RNA biology, including transcription, splicing, polyadenylation, modification, stabilization, localization, and translation (13). Moreover, competition between pairing-intronic sequences may allow generation of several circRNAs from the same gene (10). Conventional splicing signals and the speed of RNA polymerase II–mediated transcription may interfere with and regulate circRNA biogenesis (14). Arthrobacter luteus (ALU) sequences with over 1 million insertions make up more than 10% of the human genome (15). In the flanking intron, from exons, inverted repeated ALU sequences (IRALUs) are also involved in regulating the majority of human circRNA biogenesis by back-splicing (14) (Figure 1). Some RBPs also act in the biogenesis of circRNAs. Thus, the immune factors, nuclear factor 90 (NF90) and NF11, promote circRNA formation in the nucleus by directly binding IRALUs in the context of viral infection (16). Moreover, adenosine deaminase RNA specific 1, an enzyme responsible for binding dsRNA and converting A to I by deamination (17), and the abundant DExH-box helicase 9–encoded nuclear ATP-dependent RNA helicase A (18), antagonize circRNA circularization by destabilizing IRALU-mediated RNA pairing. Finally, emerging evidence has shown an important role of histone modifications in circRNA biogenesis. H3K36me3 enrichment (also involved in alternative splicing) at the 5′ circularizing exons (19) and the role of this histone mark in circRNA biogenesis (recently identified by machine learning) (20) emphasize the link between alternative splicing and circRNA biogenesis. In contrast, H3k79me2 and H4k20me1 marks were reported as negative regulators of circRNA expression (20).

## CircRNA characteristics

Large-scale RNA profiling has indicated that approximately 75% of the human genome can be transcribed into RNA (21). Twenty percent of genes produce circRNAs in the human brain, but only 9% do so in the heart (22). Moreover, because some circRNAs have higher expression levels in low-proliferating cells compared than high-proliferating cells, the abundance of circRNAs seems to be cell type specific (23). Most circRNAs are generated from pre-mRNAs, which also produce linear RNA forms, at a similar level of expression (24). Although circRNAs are abundant, they are generally expressed at low levels compared with their host mRNAs (25), and circRNA expression does not always correlate with the expression of the parental linear mRNA. Indeed, circRNAs can sometimes be expressed at much higher levels than their linear counterparts (26, 27). CircRNAs are evolutionarily conserved across species and present in most organisms, including archaea, plants, yeast, and most metazoans (28). They also have specific subcellular localizations. Thus, while some circRNAs, such as ciRNAs and EIcircRNAs, are retained in the nucleus, most circRNAs accumulate in the cytoplasm (29). Nuclear export of circRNAs uses two separate pathways based on length. Most circRNAs, generated from exon(s), are exported to the cytoplasm. Factors required for export are determined by the size of the circRNA. In human cells, the ATP-dependent RNA helicase UAP56 regulates nuclear export of long circRNAs, and URH49 is required for nuclear export of short circRNAs (30, 31). Moreover, the N6-methyladenosine (m6A) modification promotes cytoplasmic export of circRNAs. Indeed, m6A-modified circRNA in the nucleus could be recognized by the RNA m6A reader YTHDC1 and exported to the cytoplasm (32). Although most circRNAs exist in the cytoplasm, they are remarkably stable and resistant to exonucleases, such as ribonuclease (RNase) R, because they lack free ends (33). Moreover, circRNAs have a longer half-life than their host linear RNA counterparts. CircRNAs have an average half-life in cells that exceeds 48 hours, while mRNAs only last for 10 hours on average (34). This feature makes circRNAs ideal biomarkers of diseases, including cancer.

## Biological properties of circRNAs

**Figure 2:**
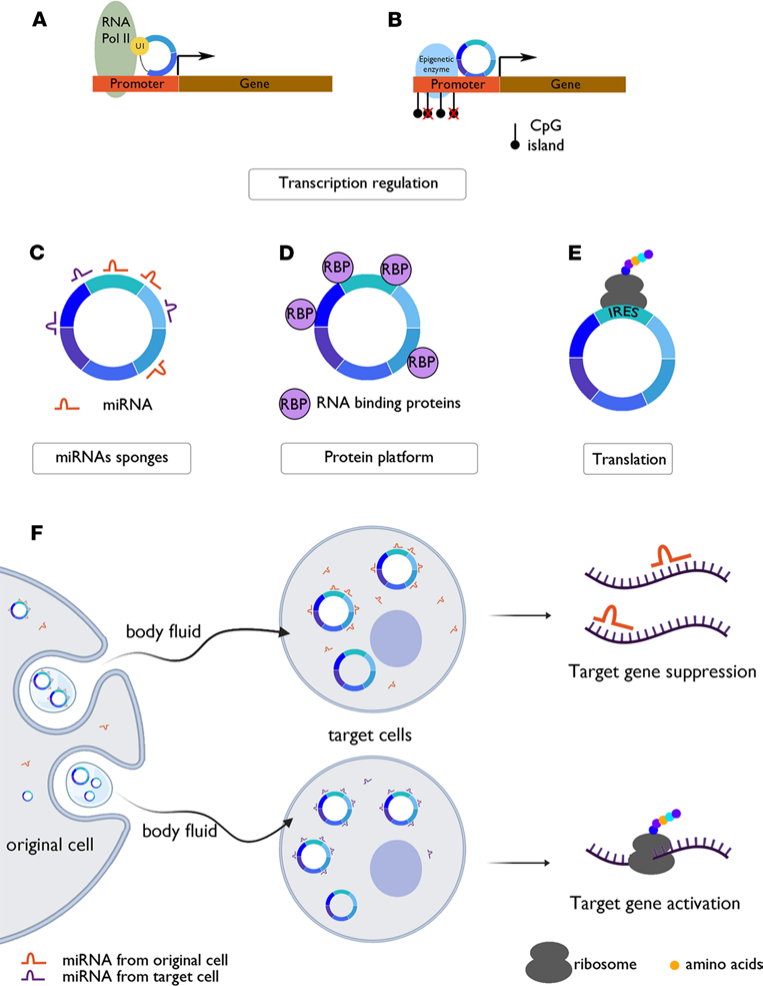
CircRNA functions. (**A**) Splicing competitors between back-splicing for circRNAs and canonical splicing for linear RNAs. Multiple circRNAs and linear RNAs can be generated from a single gene locus. Indeed, RNA pairing formed across flanking introns promotes back-splicing, leading to the formation of a circRNA and a linear RNA with exon exclusion, whereas RNA pairing formed within one individual intron promotes canonical splicing, inducing a linear RNA with exon exclusion but not back-splicing. Thus, the competition between these reverse complementary sequences can lead to multiple circRNAs. (**B**) Transcription regulators. CircRNAs can interact with RNA pol II, the complex containing U1 snRNP, and enhance gene transcription. CircRNAs can also silence genes by regulating parental genes through epigenetic mechanisms. (**C**) MiRNA sponges. CircRNAs can bind to miRNAs, thus inhibiting miRNA-mRNA conjugation. (**D**) Protein sponges. CircRNAs can restrain RBP from binding to miRNA or mRNA, thus indirectly regulating the protein biogenesis and the function of RBP. (**E**) Protein templates. CircRNAs can be translated into peptides when equipped with IRESs and AUG site(s). (**F**) Packaged into exosomes. Exo-circRNAs are circRNAs in exosomes where they can bind to miRNAs (top). After entering target cells, miRNAs are released and target genes can be silenced. In contrast, when exo-circRNAs are not bound to miRNAs in exosomes (bottom), they are able to sponge specific miRNAs in target cells and target genes are activated. U1, U1 small nuclear ribonucleoprotein particle (snRNP); RNA pol II, RNA polymerase II; RBP, RNA-binding protein; IRES, internal ribosome entry sites.

CircRNAs have diverse functions in human cells. Some circRNAs have exhibited the capacity to regulate both distinct targets and their own parental gene. CircRNAs may regulate nuclear gene expression by interfering with splicing (Figure 2A) or transcription processes, via epigenetic modulation (Figure 2B), acting as miRNA sponges through miRNA response elements (MREs) (Figure 2C), interacting with RBPs (Figure 2D), being translated into proteins or peptides (Figure 2E), or being exported outside the cell through exosomes (Figure 2F).

### CircRNAs interfere with nuclear mechanisms of gene expression.

CiRNAs and EIcircRNAs (i.e., intron-retaining circRNAs) are primarily located in the nucleus and can therefore regulate gene expression by interfering with alternative splicing events or the transcription process through binding and modulating activity of RNA polymerase II and U1 snRNP (35, 36). CircRNAs may also epigenetically regulate expression of their parental gene by inducing demethylation or maintaining DNA methylation at promoter CpG islands through recruitment of ten-eleven translocation methylcytosine dioxygenase 1 (TET1) demethylase or DNA methyltransferase 1, which is critical for maintaining DNA demethylation during DNA replication (10, 37)

### CircRNAs act as miRNA sponges.

As EcircRNAs are mostly located in the cytoplasm, they may promote postnuclear regulation of gene expression. Many EcircRNAs harbor sequences complementary to MREs and thus may act as miRNA sponges. MRE-containing circRNAs have the capacity to sequester corresponding miRNAs, thus precluding their binding to target mRNAs and enhancing target gene expression. Conversely, circRNA downregulation may free miRNAs and inhibit gene expression (38, 39). This function was first identified in murine sex-determining region Y, which is responsible for mammalian sex determination and produces a circRNA with 16 miR-138–binding sites (40). In human, ciRS-7 (also known as cerebellar degeneration-related protein 1 antisense, CDR1as) consists of a single exon and possesses 74 MREs for miR-7. CiRS-7 can also bind argonaute proteins that can interact with miRNAs (41, 42). Of note, some circRNAs share the same MREs with the 3′-UTR sequence of the parental transcript, thus upregulating the parental gene through sponging these miRNAs (10).

### CircRNAs bind to RBPs.

RBPs can be classified into two categories: 1) those interacting with sequence-specific motifs of particular mRNAs and 2) those interacting with elements, such as the cap and poly(A) tail, common to many mRNAs.

### CircRNAs can act as a decoy or cargo for RBPs.

Indirect or direct interactions between circRNA and RBPs encoded by the parental gene are possible (43). With the help of emerging and developed web tools, interactions of circRNAs with miRNAs and RBPs can be explored, and thus circRNA networks can be predicted (21, 44).

### Translation into proteins or peptides.

In eukaryotic cells, the mRNA 5′ cap and 3′ poly(A) tail are essential for translation, as they bind to translation initiation factors that facilitate ribosome recruitment. Due to the absence of 5′ and 3′ ends, circRNAs were initially considered ncRNAs. However, certain mRNAs can be translated via an alternative cap-independent mechanism because of specific sequences termed IRESs that are generally located in the mRNA 5′-UTR. Studies have shown that circRNAs can associate with polysomes, and some circRNAs include an AUG initiation codon and a putative ORF (45), suggesting protein-coding potential in IRES-containing circRNAs. Accordingly, a recent study showed that the *ZNF609* circRNA encompasses most of the *ZNF609* mRNA 5′-UTR, which serves as an IRES allowing translation of the circRNA into a truncated ZNF609 protein (46). Translation of circRNAs has also been shown to be enhanced by m6A. For *ZNF609* circRNA, translation is enhanced through m6A-mediated recruitment of the cap-independent translation initiation factor eIF4G2 (47, 48). Some reported circRNA-encoded peptides display significant antitumor functions by interfering with the proliferation and metastasis abilities of solid cancers (45).

### Export of circRNAs through exosomes.

Exosomes are small vesicles secreted by most cells and have important functions in cellular communication, especially between distant cells (49). Exosomes can contain DNA, proteins, lipids, and different types of RNA, including mRNAs and miRNAs (50). In cancer, exosomes are reportedly involved in metastasis and contribute to preparation of the metastatic niche (51). Exosomes are also cell type specific and released into biofluids, such as blood, urine, and saliva, making them promising biomarkers (52). The presence of circRNAs in exosomes was first reported in 2015 (53), and this class of circRNAs was termed “exo-circRNAs.” RBPs, such as heterogeneous nuclear ribonucleoproteins A2/B1, or HNRNPA2B1, can bind and transfer miRNAs into exosomes through binding exosome motifs, which are enriched in exosomes but not in cells (54). In the exosome, circRNAs conserve miRNA-sponging activity and can modulate miRNA target gene expression in the recipient cell (Figure 2E) (55). CDR1as is the first circRNA reported to have a biological function in exosomes and can suppress growth in recipient cells by sponging miR-7 in the exosome (53). In addition, exosomal circPDE8A release enhances, via the miR-338/MACC1/MET pathway in pancreatic cancer, tumor invasiveness through exosome-mediated cell communication (56). Thus, circRNA-containing exosomes can inhibit or promote cancer progression. Moreover, higher expression of circRNA in exosomes was revealed via analysis of liver cancer cells and cell-derived exosomes through RNA sequencing. Interestingly, using the same liver cancer cell line in a murine xenograft model, Li et al. correlated exo-circRNAs’ expression levels with tumor mass (53). This pioneering study opened a new research field, and exo-circRNAs have since been detected in several cancer entities, including serum samples of patients diagnosed with gastric cancer, hepatocellular cancer, and pancreatic cancer (57–59). In all these studies, exo-circRNA abundance correlated with advanced clinical stage and metastasis. Together, these studies have shown circRNAs to be enriched in cancer cell–derived exosomes and to be relatively stable in biofluids because of their ringlike structure, making exo-circRNAs promising future biomarkers for detection by noninvasive liquid biopsies.

## Discovering and profiling circRNAs

Despite the discovery of more than 100,000 unique human circRNAs, these molecules are understudied compared with other types of transcripts. Most public RNA-sequencing data sets have been generated from RNAs isolated through their poly(A) tails, thus excluding circRNAs. Moreover, the apparent quantity of circRNAs found in RNA-sequencing data sets is certainly underestimated because of biases created by the large amount of rRNA. Yet, due to evolution of alternative RNA-sequencing and microarray technologies, the study of circRNAs’ structure and function is becoming easier. Indeed, genome-wide profiling of circRNAs must use alternative methods for depleting rRNA (for example, Ribo-Zero technology) rather than or in combination with poly(A) depletion. CircRNA identification may also be improved by treating RNA samples with exonuclease RNase R to degrade linear RNAs, thus enriching for circRNA (21). However, RNA sequencing of rRNA-depleted total RNA has been the preferred method for the discovery of novel circRNAs. This approach offers the advantage of simultaneously providing expression data for both coding and ncRNAs. Depending on the expression level of circRNAs in the studied tissues, at least 100 bp sequencing is recommended to obtain sufficient read lengths to allow accurate prediction of circRNAs based on read-spanning back-splicing junctions (BSJs). Microarrays are possible alternatives to RNA sequencing for circRNA identification and quantification. Arraystar Inc. has introduced the first commercially available microarrays for human, mouse, and rat circRNAs. The arrays include more than 10,000 circRNA templates selected from key publications. The workflow includes RNase R treatment. Although this treatment increases the assay accuracy, it prevents assessment of the ratio of linear to circRNA in cases where both types of transcripts can be produced from the same gene (60). Despite the advantage of requiring much less bioinformatics, the microarray strategy is based on previously described circRNAs, thus precluding detection of novel circRNAs.

Alternatively, NanoString assays generate reproducible and quantitative results that are potentially more reliable than those obtained from RNA sequencing, as no RNA PCR amplification is needed. As has been shown, reverse transcription or PCR can introduce a bias and lead to artifactual circRNA amplification (61). Indeed, NanoString technology is a digital counting method that delivers results quickly and does not require bioinformatic expertise. Moreover, data can be obtained in a clinical setting from severely degraded RNA samples from formalin-fixed, paraffin-embedded (FFPE) tissues (as already used in clinics for mRNA isolated from patient FFPE tissue sections; ref. 62). Unfortunately, it is not suitable for biomarker discovery, as it employs approximately 800 preselected genes based on publication data.

New bioinformatic algorithms are continuously being developed and are now available for identifying and quantifying circRNAs from RNA-sequencing data. To date, the CIRI2 algorithm has the highest sensitivity for circRNA detection but requires checking with two independent algorithms to ensure BSJs are correctly annotated. Indeed, sequence homology and degenerated sequences at exon boundaries may interfere with circRNA prediction. Furthermore, the mapper (e.g., STAR) and associated stringency requirements may vary between algorithms, and their false-positive rates differ substantially. Thus, in the absence of a gold standard algorithm, running more than one circRNA prediction algorithm can minimize the number of false positives (21).

## Analysis of circRNA interactions using web tools

CircRNA databases (Table 1) have been developed to explore circRNA partners and are excellent platforms to predict miRNA- or RBP-circRNA interactions. StarBase v2.0 is the first database to allow systematic identification of regulatory RNA-RNA interaction networks using experimental Cross-Linking ImmunoPrecipitation sequencing data (63). This database has identified approximately 9000 miRNA-circRNA regulatory interactions. The CircInteractome web tool is the first web tool developed to predict miRNA-binding sites on circRNAs and thus may identify potential sponged miRNAs that are crucial for posttranscriptional gene regulation (6, 64). CircBase contains publicly annotated circRNA data sets and can be used to check whether circRNAs are defined and characterized in public databases (65). CircFunBase is a repertoire of functional circRNAs that provides both experimentally validated and computationally predicted functions of identified circRNAs (66). Cancer-Specific CircRNA Database collects circRNAs from tumor cell lines and normal cells from Encyclopedia of DNA Elements and predicts chromosomal localization of circRNAs, MREs, RBP binding sites, and variable splicing of related genes (67). Finally, circad is a database of disease-associated circRNAs that includes PCR primer assay lists. To date, circad is the most comprehensive database of disease-associated circRNAs (68).

## CircRNAs as potential cancer therapeutic targets or biomarkers

Consistently, some circRNAs have been shown to play relevant roles in normal development of tissues and organs such as skeletal (e.g., circZNF609, circBNC2) (46, 69) and cardiac muscles (70), in neural systems (71), in aging (72), in the maintenance of pluripotency (e.g., circBIRC6) (73), in the immune system (e.g., circ-0032139 from *HIF1A* gene and circZC3H4) (74, 75), and in human pathologies, such as Alzheimer disease (ciRS) (76), Parkinson disease (circCDR1as) (77), diabetes (hsa_circ_0068087, hsa_circ_0124636, hsa_circ_0139110, and hsa_circ_0018508), and cancers (78). CircRNAs can promote oncogenic features, such as cell cycle completion, tumor growth, and invasiveness, as well as epithelial-mesenchymal transition (EMT; e.g., circPTPN22; ref. 79). Notably, in EMT, circRNAs can play bivalent roles: leading to EMT inhibition through inactivation of NOTCH, TGF-β and β-catenin pathways (80–82) or to EMT promotion through the activation of WNT, AKT/mTOR, and TWIST1 pathways (83–85). While some circRNAs seem to be specific to one cancer type, others have demonstrated activities in different cancers. For example, circFOXO3 is involved in at least 6 cancers (prostate, bladder, esophageal squamous cell, urothelial carcinoma, breast, and non-small cell lung cancers) (59, 86–89). Likewise, circHIPK3 is overexpressed in 6 different cancer types (prostate, liver, breast, colon, bladder, and stomach) (90, 91) and involved in both cell proliferation and autophagy regulation (92). Importantly, circRNAs can be targeted by siRNAs to potentially block their carcinogenic activity, thus making them good candidates for anticancer therapies. Moreover, circulating circRNAs can be detected in almost all human body fluids and secretions, including blood, breast milk, saliva, urine, and cerebrospinal fluid, and are stable and RNase resistant (Figure 2); therefore, they could be considered potential cancer biomarkers in the clinic for early diagnosis and prognosis (93).

## CircRNAs as biomarkers in hematological cancers

### CircRNAs and leukemia.

Supplemental Table 1 (supplemental material available online with this article; https://doi.org/10.1172/jci.insight.151513DS1) summarizes circRNAs involved in hematological diseases. High-throughput sequencing and microarray technologies have been used for comparative analyses of circRNAs between PBMCs or BM from patients with acute myeloid leukemia (AML) and controls for different settings: 1) with and without extramedullary infiltration (94, 95), 2) with WT and mutated nucleophosmin 1 (*NPM1*) (96), 3) positive or negative for Fms-related receptor tyrosine kinase 3–internal tandem duplication (FLT3-ITD) mutations, and 4) in doxorubicin-sensitive and -resistant AML cell lines (97, 98). In vitro functional investigations using AML cell lines showed that circ-0004136 and circ-0009910 (circularized transcript of mitofusin 2, *MFN2*) promote cell proliferation and growth of AML cells by dysregulating miR-142 and miR-20a-5p, respectively (99, 100). These data were further confirmed in vivo for circ-0009910 (99). Circ-0001947 targets the miR-399-5p/CREB3 regulatory factor (CREBRF) axis, curbing cell proliferation (101), whereas circ-100290 (circularized transcript of solute carrier family 30 member 7, *SLC30A7*) promotes cell proliferation and restrains apoptosis through the miR-203/Ras-related protein Rab10 axis (102). In nude model mice, circ-0001947 inhibits AML cells’ growth (101). In de novo patients with AML, upregulation of a circular form of vimentin mRNA appears to predict poor clinical outcomes and is associated with shorter overall survival (103). In addition, circ-0004277 (circularized transcript of WD repeat domain 37, *WDR37*) is increased in relapsed and refractory patients (95). Moreover, by sponging miR-181, overexpression of circANAPC7 (also known as hsa_circRNA-101141) promotes tumorigenicity (94). CircRNA-DLEU2 (also known as circ-0000488) is expressed in AML tissues and stimulates cell proliferation in C57BL/6 mice by suppressing miR-496 expression and stimulating protein kinase CAMP-activated catalytic subunit β (*PRKACB*) transcription in AML cell lines (103). CircPAN3 is upregulated in refractory AML patients and in doxorubicin*-*resistant AML cell line THP-1. By sponging miR153-5p and miR183-5p, circPAN3 decreases the antiapoptotic X-linked inhibitor of apoptosis expression and mediates doxorubicin resistance in THP-1 cells (97). Due to their stability, circRNAs may better serve as potential biomarkers for AML classification and risk stratification than their linear counterparts. For example, circ-0075001 expression positively correlates with expression of total linear *NPM1* mRNA but is independent of the *NPM1* mutational status in AML patients (96). In addition, circRNAs were recently shown to modulate cell proliferation independently of their related linear RNAs (104). Circ-0121582, a reverse splicing product of the glycogen synthase kinase 3β (GSK3β) exon 1 to exon 7, suppresses AML cell growth in vitro and in vivo. The circ-0121582/miR-224/GSK3β axis forms in the cytoplasm and binds the *GSK3B* promoter to recruit TET1 in the nucleus (105). In FLT3-ITD–positive AML cell lines, circMYBL2 interacts with the RBP polypyrimidine tract-binding protein 1 to impair AML cell proliferation and overcome acquired resistance to FLT3 tyrosine kinase inhibitor quizartinib. Of note, knockdown of circMYBL2 decreases phosphorylation of FLT3 and STAT5, the downstream FLT3 target crucial for AML progression in NOD/SCID mice (98). Circ-0000370 was also recently shown to be abnormally expressed in FLT3-ITD–positive AML patients and cell lines and is derived from the *FLI1* gene. In vitro, circ-0000370 promotes viability and suppresses apoptosis of FLT3-ITD–positive AML cells through modulation of miR-1299 and S100 calcium-binding protein A7A expression (106). More recently, total RNA sequencing in younger adults with de novo, cytogenetically normal AML (pretreatment BM or blood samples from 365 adult patients <60 years of age) revealed that circFBXW7 acts as a tumor suppressor (107).

In chronic lymphocytic leukemia (CLL) patient samples and cell lines, transcriptomic sequencing revealed that circ-0132266 was involved in tumorigenesis by interacting with miR-337-3p to modulate promyelocytic leukemia protein (PML) expression (108), whereas circ-CBFB contributed to CLL progression by modulating the miR-607/FZD3/Wnt/β-catenin cascade (109). Microarrays were also used to study plasma samples from three patients with CLL and five healthy individuals. Seventy-one circRNAs were differentially expressed, and circRPL15 was the most markedly upregulated in the plasma of patients and positively correlated with a poor disease prognosis, suggesting circRPL15 as a new class of plasma diagnostic biomarker in CLL. In vitro, circRPL15 indirectly regulates the RAS/RAF1/MEK/ERK pathway to increase CLL cell viability through binding to miR-146b-3p (110). In chronic myeloid leukemia (CML), high-throughput sequencing and microarray technologies on PBMCs or patient BM identified two circRNAs involved in CML pathogenesis. Thus, the circHIPK3 (also referred to as circ-0000284) levels in PBMCs and patient sera were significantly higher than in healthy individuals and positively correlated with poor prognosis (90). Moreover, hsa_circ_0080145 was significantly upregulated in CML PBMCs and serum samples compared with healthy controls. This circRNA promotes CML progression by acting as a sponge for miR-29b, which plays a tumor-suppressive role by targeting both ABL proto-oncogene 1 (ABL1) and breakpoint cluster region protein–ABL (BCR-ABL) protein expression (111). In nude model mice, circ_0080145 silencing inhibits BCR-ABL tyrosine kinase inhibitor imatinib resistance (112). Additionally, an association between circ-100053 overexpression and imatinib resistance has been suggested for patients with CML (113).

In BM from patients with acute lymphoblastic leukemia (ALL), high-throughput sequencing quantitative reverse transcription PCR (qRT-PCR) identified circPVT1 (also known as circ-0001821), circPAx5, and circHIPK3 as significantly upregulated in children with B cell acute lymphoblastic leukemia (114). CircPVT1 expression was higher in the BM of patients with ALL than in specimens from healthy individuals. CircPVT1 sponges miR-let-7 and miR-125, of which the respective targets are MYC and B cell lymphoma 2 (BCL2). In Nalm-6 and Jurkat cells, knockdown of circPVT1 inhibits both BCL2 and MYC expression. Inhibition of circPVT1 could therefore interfere with cell proliferation by inducing apoptosis, suggesting circPVT1 as a potential therapeutic strategy (115). Recently analysis of RNA-sequencing data from patients with T cell acute lymphoblastic leukemia, or T-ALL, and from thymocyte populations of healthy human donors defined subtype-specific circRNA signatures in molecular genetic T-ALL subgroups. Specifically, circZNF609, circPSEN1, circKPNA5, and circCEP70 were upregulated in immature T-ALL; circTASP1, circZBTB44, and circBACH1 associated with the TLX3 rearrangement; circHACD1 and circSTAM were prevalent in homeobox A cluster (HOXA); and circCAMSAP1 and circCASC15 associated with TLX1 TAL-LMO rearrangements. These results were validated in patient tissues and in a panel of 13 cell lines. Functional analysis revealed that in vitro silencing of circZNF609 decreases cell viability in T-ALL cell lines. Moreover, a circRNA-miRNA gene interaction network outlined interactions between circZNF609 and miR181 in immature T-ALL subtypes, circZBTB44 and miR-let-7i-5p in TXL3, and circHACD1 and miR-182-5p in HOXA (116).

### CircRNAs and multiple myeloma.

In BM plasma cells from patients with multiple myeloma (MM), healthy controls, and cell lines, sequencing and microarray technologies identified significant downregulation of circSMARCA5. Patients with high circSMARCA5 expression have a higher complete response rate, longer progression-free survival, and improved overall survival, highlighting the potential of this specific circRNA as a diagnostic marker. In vitro functional experiments in MM cell lines show that overexpression of circSMARCA5 inhibits MM cell proliferation and promotes apoptosis by sponging miR-767-5p (117). In MM cell lines, hsa_circ_0007841/SEC61A1 enhances chemotherapy resistance through upregulating the ATP-binding cassette transporter G2 (ABCG2) (118). In BM samples collected from patients with MM as compared with healthy controls, circPTK2 and circAFF2 were shown to be upregulated and downregulated, respectively. CircPTK2 enhances expression of its parental oncogene, protein tyrosine kinase 2 (PTK2). Moreover, by sponging miR-1298-5p, which has tumor-suppressive activity, circPTK2 promotes tumor progression. CircAFF2 sponges miR-638, inhibiting its oncogenic function. Combined circPTK2 and circAFF2 expression correlates with poor treatment response and survival, whereas expression of circAFF2 alone associates with a better prognosis (119). Moreover, circRNA circ_0000190 inhibits the progression of MM through modulating miR-767-5p/MAPK4 pathway in vitro and inhibits growth of human MM tumor in NOD/SCID xenograft models (120). Finally, knockdown of circCDYL/miR-1180/YAP hinders MM growth in nude xenograft models (121).

### CircRNAs and lymphomas.

So far, few studies have evaluated circRNA expression profiling in connection with lymphoma. Using NanoString technology, 52 unique circRNAs were quantified in cell lines and patient samples from several different B cell malignancies (mantle cell lymphoma, MM, diffuse large B cell lymphoma [DLBCL], follicular lymphoma, Burkitt lymphoma, and CLL). The circRNA expression profiles obtained could distinguish different B cell malignancies and confirmed the presence of a novel host IKAROS family zinc finger 3–derived (*IKZF3-*derived) circRNA (62).

Moreover, a circRNA microarray was used to compare and analyze expression profiles of three pairs of DLBCL tissues and adjacent tissues and identified circAPC (hsa_circ_0127621), derived from exon 7 to exon 14 of adenomatous polyposis coli (APC) gene, in DLBCL tissues, plasma, and cell lines. In patients with DLBCL, qRT-PCR validated markedly lower plasma circAPC levels. Moreover, by sponging and inhibiting miR-888, cytoplasmic circAPC increases APC expression by binding the *APC* promoter and recruiting DNA demethylase TET1. Further, circAPC inhibits proliferation of DLBCL that has been subcutaneously introduced in nude xenograft model mice. Functional experiments revealed that circAPC overexpression in U2932 and TMD8 cells inactivates Wnt/β-catenin signaling via miR-888 and the TET1/APC axis, suggesting that the restoration of circAPC expression has a potential therapeutic effect on DLBCL. Of note, *APC* mRNA expression was downregulated in DLBCL cell lines and tissues and positively correlated with circAPC expression, suggesting this as a clinically useful marker of DLBCL diagnosis and prognosis (122). In parallel, a recent study using RNA sequencing provided a landscape of circRNA expression for several mantle cell lymphoma (MCL) cell lines. Most of the identified circRNAs are exonic and are expressed at a lower level than the corresponding host genes. Some circRNA host genes known to play a role in lymphoma development can produce only a single circRNA, whereas others, such as *ATM*, *WHSC1*, and *XPO1*, can generate multiple circRNAs (62).

Despite decades of effort, therapeutic strategies against T cell lymphoblastic lymphomas (T-LBLs) remain inefficient because of lack of effective markers to detect tumor recurrence and metastasis. CircRNA microarray to comparatively analyze circRNA expression profiles of three pairs of T-LBL and normal pediatric thymus tissues led to the identification of a large number of differentially expressed circRNAs. This analysis revealed that circLAMP1 was markedly upregulated in cell lines and samples from T-LBL patients. The lysosomal associated membrane protein 1–encoded (*LAMP1*-encoded) protein is a member of a membrane glycoprotein family and may play a role in tumor cell metastasis. Functional in vitro experiments using cell lines confirmed that circLAMP1 sponges miR-615-5p, thereby indirectly regulating discoidin domain receptor 2 (DDR2) expression, inhibiting T-LBL cell apoptosis, and promoting cell proliferation. DDR2 belongs to the family of receptor tyrosine kinases that bind to and are activated by collagen in the extracellular matrix. Recently, DDR2 has been implicated to drive proliferation and metastasis in a number of cancer types (123).

## Fusion circRNAs expressed from translocation-associated cancers

**Figure 3:**
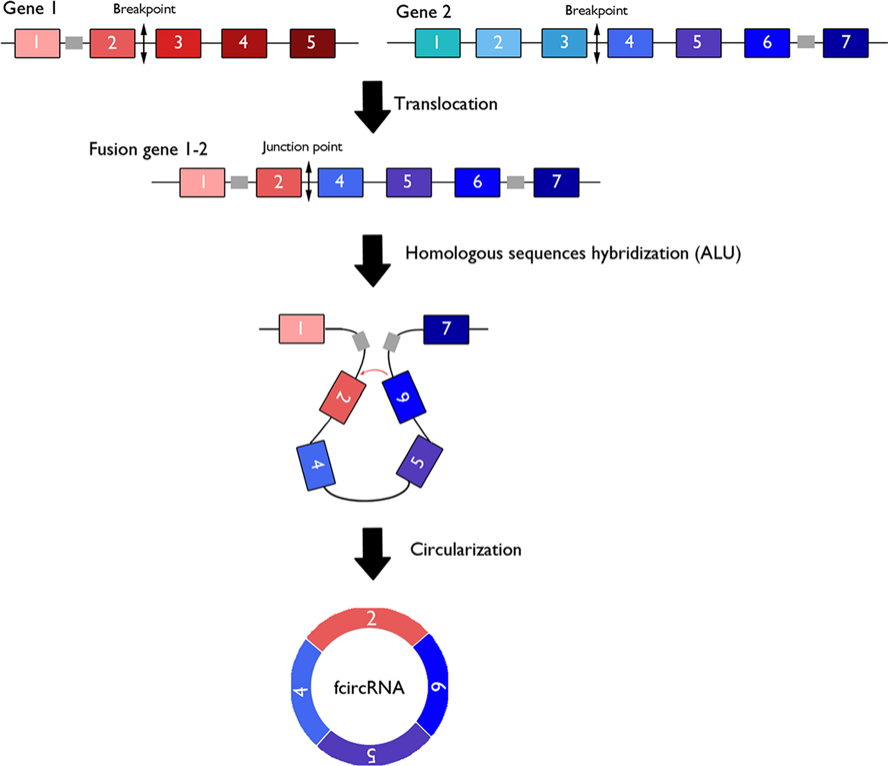
Fusion circular RNAs derived from chromosomal translocations. Transcription of fusion genes generated by cancer-associated chromosomal translocation can generate both linear mRNAs coding for oncogenic fusion proteins and fusion circular RNAs (f-circRNAs) via cis-acting factors such as repeated ALU pairs.

Oncogenic gene fusion is common in cancer samples and plays a crucial role in carcinogenesis. Fusion circRNAs (f-circRNAs) are generated from chromosomal rearrangements, which could promote back-splicing of chimeric mRNA transcripts, derived from chromosomal translocations. This event could be triggered by a repetitive sequence flanking the breakpoint region in the pre-mRNA transcript (Figure 3) (124). Ultra-deep RNA sequencing of various cancer samples reveals the expression of f-circRNAs from fusion mRNAs in non-small cell lung cancers, Ewing sarcomas, and hematological malignancies (125, 126). To date few f-circRNAs have been described in the literature and have mainly been described in hematological cancers.

### F-circRNAs and acute promyelocytic leukemia.

The *PML*/retinoic acid receptor α (*PML/RARA*) fusion gene originates from the t(15;17) (q24;q21) translocation and is the most recurrent chromosomal rearrangement in acute promyelocytic leukemia (APL). In APL patient biopsies and in the NB4 APL-derived human leukemic cell line, the *PML/RARA* transcript could generate one or two f-circRNAs, called f-circPR. One f-circPR isoform, with a back-splicing junction joining PML exon 5 and the RARα exon 6, was present in all analyzed patients and in the NB4 cell line, while the f-circPR isoform that harbors a back-splice junction between PML exon 4 and RARα exon 4 was found in the NB4 cell line but not in all patients (124).

### F-circRNAs and AML.

In AML, the t(9;11) (p21;q23) translocation generates different isoforms of the recurrent mixed lineage leukemia (MLL)/AF9 (KMT2A/MLLT3) fusion transcripts. Two f-circRNAs, *f-circM9*s (*f-circM9_1* and *f-circM9_2*), were identified in THP-1 cells. The back-splice junction between the 5′ head of *MLL* exon 7 and the 3′ tail of *AF9* exon 6 formed f-circM9_1, while f-circM9_2 displayed its back-splice junction between the 5′ head of *MLL* exon 5 and the 3′ tail of *AF9* exon 6. Investigations on f-circPR and *f-circM9_1* in immortalized mouse embryonic fibroblasts revealed that these f-circRNAs promote cell proliferation, reduce apoptosis, and play roles in treatment resistance, thus favoring leukemia progression both in vitro and in vivo and independent of the linear transcript and fusion protein counterparts (124). Similarly, the circRNA from the different fusion of MLL-AF4 also plays an oncogenic role in AML and promotes leukemogenesis in NOD/SCID mice (127). It was recently suggested that circRNA expression can be affected by the type of fusion and the breakpoint position, which in turn influences the expression level or activates cryptic back-splicing sites on circRNAs that are normally generated by partner genes (128). Since *MLL* can fuse with over 90 partner genes, it is prone to give rise to different circRNAs with distinct functions.

### F-circRNAs and CML.

In CML, f-circRNA *circBA9.3*, which results from a juxtaposition of *ABL1* exon 3 to *BCR* exon 9, was detected in peripheral blood cells from patients with CML harboring the t(9;22) (q34;q11) translocation that results in the *BCR/ABL1* fusion gene. *CircBA9.3* overexpression in leukemic cell lines promotes proliferation and decreases apoptosis, by improving BCR/ABL1 translation or preventing its degradation. In addition, *circBA9.3* increases both ABL1 and BCR-ABL1 expression and reduces sensitivity of cancer cells to imatinib, suggesting that it is involved in CML resistance (129).

### F-circRNA in anaplastic large cell lymphoma.

In anaplastic large cell lymphoma (ALCL), oncogenic NPM/ALK signaling is mediated by several pathways, which play major roles in lymphomagenesis by controlling key cellular processes such as cell cycle progression (130, 131). The t(2;5) translocation is the most frequent chromosomal translocation in ALK^+^ ALCL (84% of cases) and occurs mainly between exon 4 of *NPM* and exon 20 of *ALK* (130). The NPM-ALK translocation fuses the tyrosine kinase domain of the ALK protein to the dimerization segment of NPM1. Interestingly, we recently identify several novel f-circRNAs using a CRISPR/Cas9-based approach to induce the NPM-ALK translocation in mouse pro-B cell line BaF3 and in human htert-RPE1 cells. Importantly, these f-circNPM-ALKs were also detected in patient-derived NPM-ALK^+^ ALCL cell lines, SU-DHL1 and SUPM2 (61). We and others have also highlighted the role of miRNA in oncogenic ALK signaling in NPM-ALK^+^ ALCL. Thus, miRNAs influence the cellular phenotype and pathogenesis of NPM-ALK^+^ ALCL and are emerging as tissue-specific biomarkers with potential clinical applications for both identifying cancer subtypes and developing new therapies (132–134). f-circNPM-ALKs could also act as miRNA inhibitors in ALCL. Importantly, several f-circEML4-ALKs were identified in EML4-ALK fusion-expressing non-small cell lung cancer cell lines (125). Some of these f-circRNAs were detectable in patient tumors, but only one, f-circEA-2a, has been shown to circulate in fluids, thus providing new perspectives as to the use of other f-circRNAs and implicating the ALK kinase as a biomarker. The detection of f-circRNA-NPM-ALK could also be used as a therapeutic and/or diagnostic biomarker for NPM-ALK^+^ ALCL (126). To date, the presence of these NPM-ALK translocation-derived f-circRNAs in patient biopsies and their role in ALCL tumorigenesis remains to be investigated.

## Conclusions and perspectives

The biochemical properties of circRNAs make them attractive for potential clinical applications. First, due to their potential stability in body fluids, they may represent good biomarkers accessible by noninvasive sampling; however, more work must be done before implementation. In particular, retrospective and, above all, prospective clinical trials have to be conducted before any use in clinical practice. Furthermore, circRNAs and f-circRNAs may face distinct challenges as applicable biomarkers. Indeed, although the expression of standard circRNAs may vary from one group of patients to another, it remains challenging to set precise thresholds capable of distinguishing two groups. Threshold determination might be rendered a bit easier thanks to the use of signatures that include a sufficient number of different circRNAs, as circRNAs are likely not to be in limited numbers. It can be therefore anticipated that the use of circRNA signatures should ensure a good sensitivity for clinical tests. The challenge appears quite different for f-circRNAs, as only a few are expected to be present in a given pathology. The limited presence of f-circRNAs provides a strong advantage, ensuring excellent specificity, and gene translocations are not expected to be found in noncancerous cells. The probable low number and low expression level of f-circRNAs may render detection quite challenging. Thus, circRNAs and f-circRNAs show different, yet complementary, characteristics whose combination could offer a real clinical interest. However, direct clinical applications of circRNA detection in liquid biopsies still appears as a challenge. Notably, circRNAs could be difficult to detect due to their low abundance in both tumors and body fluids, urging the development of very sensitive detection methods. Last, the detection in blood cells of circRNAs has potential to be more expensive and time-consuming than existing protein tests, which may limit the wide application of circRNAs as biomarkers in the short term. However, progress in high-throughput techniques will doubtlessly help identify any promising circRNAs as biomarkers.

A second potential clinical application is the use of circRNAs as therapeutic targets to be extinguished (those with oncogenic function) or to be overexpressed (those with antioncogenic function). However, this necessitates the accumulation of much more knowledge about their mechanisms of action and how their expression levels can be manipulated, particularly in preclinical in vivo settings. Providing this information represents a challenge for the scientific community in the near future.

## Supplementary Material

Supplemental data

## Figures and Tables

**Table 1:**
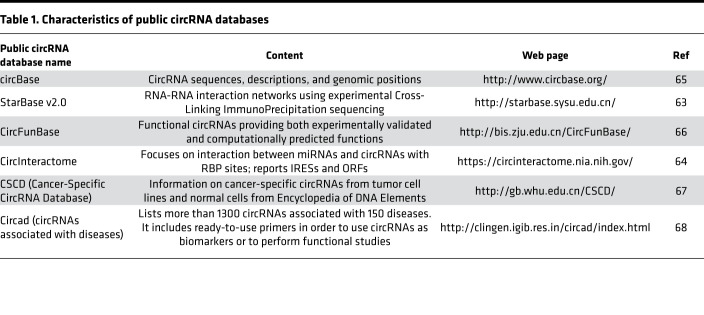
Characteristics of public circRNA databases

## Version 1 11/08/2021

Electronic publication

## References

[1] Claverie JM (2005). Fewer genes, more noncoding RNA. Science.

[2] Mattick JS (2018). The state of long non-coding RNA biology. Noncoding RNA.

[3] Mattick JS, Makunin IV (2006). Non-coding RNA. Hum Mol Genet.

[4] Lei B (2019). Circular RNA: a novel biomarker and therapeutic target for human cancers. Int J Med Sci.

[5] Chen LL, Yang L (2015). Regulation of circRNA biogenesis. RNA Biol.

[6] Panda AC (2018). Analysis of circular RNAs using the web tool CircInteractome. Methods Mol Biol.

[7] Geng X (2020). Circular RNA: biogenesis, degradation, functions and potential roles in mediating resistance to anticarcinogens. Epigenomics.

[8] Schmidt CA (2019). Molecular determinants of metazoan tricRNA biogenesis. Nucleic Acids Res.

[9] Schmidt CA, Matera AG (2020). tRNA introns: presence, processing, and purpose. Wiley Interdiscip Rev RNA.

[10] Zhao X (2019). Circular RNAs: biogenesis, mechanism, and function in human cancers. Int J Mol Sci.

[11] Zhang P (2020). Comprehensive identification of alternative back-splicing in human tissue transcriptomes. Nucleic Acids Res.

[12] Li X (2020). The mechanism and detection of alternative splicing events in circular RNAs. PeerJ.

[13] Glisovic T (2008). RNA-binding proteins and post-transcriptional gene regulation. FEBS Lett.

[14] Li Z (2015). Exon-intron circular RNAs regulate transcription in the nucleus. Nat Struct Mol Biol.

[15] de Koning AP (2011). Repetitive elements may comprise over two-thirds of the human genome. PLoS Genet.

[16] Li X (2017). Coordinated circRNA biogenesis and function with NF90/NF110 in viral infection. Mol Cell.

[17] Ivanov A (2015). Analysis of intron sequences reveals hallmarks of circular RNA biogenesis in animals. Cell Rep.

[18] Aktas T (2017). DHX9 suppresses RNA processing defects originating from the Alu invasion of the human genome. Nature.

[19] Coscujuela Tarrero L (2018). Luminal breast cancer-specific circular RNAs uncovered by a novel tool for data analysis. Oncotarget.

[20] Zhang M (2020). Revealing epigenetic factors of circRNA expression by machine learning in various cellular contexts. iScience.

[21] Chen L (2021). The bioinformatics toolbox for circRNA discovery and analysis. Brief Bioinform.

[22] Aufiero S (2019). Circular RNAs open a new chapter in cardiovascular biology. Nat Rev Cardiol.

[23] Bachmayr-Heyda A (2015). Correlation of circular RNA abundance with proliferation--exemplified with colorectal and ovarian cancer, idiopathic lung fibrosis, and normal human tissues. Sci Rep.

[24] Liang D (2017). The output of protein-coding genes shifts to circular RNAs when the pre-mRNA processing machinery is limiting. Mol Cell.

[25] Jeck WR (2013). Circular RNAs are abundant, conserved, and associated with ALU repeats. RNA.

[26] Guo JU (2014). Expanded identification and characterization of mammalian circular RNAs. Genome Biol.

[27] Maass PG (2017). A map of human circular RNAs in clinically relevant tissues. J Mol Med (Berl).

[28] Zhang Y (2013). Circular intronic long noncoding RNAs. Mol Cell.

[29] Qu S (2018). The emerging functions and roles of circular RNAs in cancer. Cancer Lett.

[30] Huang C (2018). A length-dependent evolutionarily conserved pathway controls nuclear export of circular RNAs. Genes Dev.

[31] Li Z (2019). The nuclear export of circular RNAs is primarily defined by their length. RNA Biol.

[32] Chen RX (2019). N6-methyladenosine modification of circNSUN2 facilitates cytoplasmic export and stabilizes HMGA2 to promote colorectal liver metastasis. Nat Commun.

[33] Suzuki H (2006). Characterization of RNase R-digested cellular RNA source that consists of lariat and circular RNAs from pre-mRNA splicing. Nucleic Acids Res.

[34] Meng S (2017). CircRNA: functions and properties of a novel potential biomarker for cancer. Mol Cancer.

[35] Huang C, Shan G (2015). What happens at or after transcription: insights into circRNA biogenesis and function. Transcription.

[36] Wang Y (2018). Circular RNAs: crucial regulators in the human body (review). Oncol Rep.

[37] Chen N (2018). A novel FLI1 exonic circular RNA promotes metastasis in breast cancer by coordinately regulating TET1 and DNMT1. Genome Biol.

[38] Liu J (2017). Circles reshaping the RNA world: from waste to treasure. Mol Cancer.

[39] Panda AC (2018). Circular RNAs act as miRNA sponges. Adv Exp Med Biol.

[40] Capel B (1993). Circular transcripts of the testis-determining gene Sry in adult mouse testis. Cell.

[41] Hansen TB (2013). Natural RNA circles function as efficient microRNA sponges. Nature.

[42] Memczak S (2013). Circular RNAs are a large class of animal RNAs with regulatory potency. Nature.

[43] Patop IL, Kadener S (2018). circRNAs in cancer. Curr Opin Genet Dev.

[44] Xiong DD (2018). A circRNA-miRNA-mRNA network identification for exploring underlying pathogenesis and therapy strategy of hepatocellular carcinoma. J Transl Med.

[45] Lei M (2020). Translation and functional roles of circular RNAs in human cancer. Mol Cancer.

[46] Legnini I (2017). Circ-ZNF609 is a circular RNA that can be translated and functions in myogenesis. Mol Cell.

[47] Di Timoteo G (2020). Modulation of circRNA metabolism by m6A modification. Cell Rep.

[48] Yang Y (2017). Extensive translation of circular RNAs driven by N6- methyladenosine. Cell Res.

[49] Gurung S (2021). The exosome journey: from biogenesis to uptake and intracellular signalling. Cell Commun Signal.

[50] Valadi H (2007). Exosome-mediated transfer of mRNAs and microRNAs is a novel mechanism of genetic exchange between cells. Nat Cell Biol.

[51] Hoshino A (2015). Tumour exosome integrins determine organotropic metastasis. Nature.

[52] Thery C (2002). Exosomes: composition, biogenesis and function. Nat Rev Immunol.

[53] Li Y (2015). Circular RNA is enriched and stable in exosomes: a promising biomarker for cancer diagnosis. Cell Res.

[54] Villarroya-Beltri C (2013). Sumoylated hnRNPA2B1 controls the sorting of miRNAs into exosomes through binding to specific motifs. Nat Commun.

[55] Seimiya T (2020). Emerging roles of exosomal circular RNAs in cancer. Front Cell Dev Biol.

[56] Li Z (2018). Tumor-released exosomal circular RNA PDE8A promotes invasive growth via the miR-338/MACC1/MET pathway in pancreatic cancer. Cancer Lett.

[57] Li J (2018). Circular RNA IARS (circ-IARS) secreted by pancreatic cancer cells and located within exosomes regulates endothelial monolayer permeability to promote tumor metastasis. J Exp Clin Cancer Res.

[58] Qiu C (2009). Epidemiology of Alzheimer’s disease: occurrence, determinants, and strategies toward intervention. Dialogues Clin Neurosci.

[59] Wang C (2019). Circular RNA circ-Foxo3 induced cell apoptosis in urothelial carcinoma via interaction with miR-191-5p. Onco Targets Ther.

[60] Li S (2019). Microarray is an efficient tool for circRNA profiling. Brief Bioinform.

[61] Babin L (2018). Chromosomal translocation formation is sufficient to produce fusion circular RNAs specific to patient tumor cells. iScience.

[62] Dahl M (2018). Enzyme-free digital counting of endogenous circular RNA molecules in B-cell malignancies. Lab Invest.

[63] Li JH (2014). starBase v2.0: decoding miRNA-ceRNA, miRNA-ncRNA and protein-RNA interaction networks from large-scale CLIP-Seq data. Nucleic Acids Res.

[64] Dudekula DB (2016). CircInteractome: a web tool for exploring circular RNAs and their interacting proteins and microRNAs. RNA Biol.

[65] Glazar P (2014). circBase: a database for circular RNAs. RNA.

[66] Meng X (2019). CircFunBase: a database for functional circular RNAs. Database (Oxford).

[67] Xia S (2018). CSCD: a database for cancer-specific circular RNAs. Nucleic Acids Res.

[68] Rophina M (2020). circad: a comprehensive manually curated resource of circular RNA associated with diseases. Database (Oxford).

[69] Di Timoteo G (2020). Circular RNAs in cell differentiation and development. Development.

[70] Jakobi T (2020). Deep characterization of circular RNAs from human cardiovascular cell models and cardiac tissue. Cells.

[71] You X (2015). Neural circular RNAs are derived from synaptic genes and regulated by development and plasticity. Nat Neurosci.

[72] Cai Y (2009). A brief review on the mechanisms of miRNA regulation. Genomics Proteomics Bioinformatics.

[73] Yu CY (2017). The circular RNA circBIRC6 participates in the molecular circuitry controlling human pluripotency. Nat Commun.

[74] Dang RY (2017). Circular RNA hsa_circ_0010729 regulates vascular endothelial cell proliferation and apoptosis by targeting the miR-186/HIF-1α axis. Biochem Biophys Res Commun.

[75] Yang X (2018). Silica-induced initiation of circular ZC3H4 RNA/ZC3H4 pathway promotes the pulmonary macrophage activation. FASEB J.

[76] Lukiw WJ (2013). Circular RNA (circRNA) in Alzheimer’s disease (AD). Front Genet.

[77] Kumar L (2017). Circular RNAs: the emerging class of non-coding RNAs and their potential role in human neurodegenerative diseases. Mol Neurobiol.

[78] Tang X (2021). Review on circular RNAs and new insights into their roles in cancer. Comput Struct Biotechnol J.

[79] Ma S (2021). As a biomarker for gastric cancer, circPTPN22 regulates the progression of gastric cancer through the EMT pathway. Cancer Cell Int.

[80] Li J (2017). Circ-104916 is downregulated in gastric cancer and suppresses migration and invasion of gastric cancer cells. Onco Targets Ther.

[81] Wang L (2018). Circular RNA hsa_circ_0008305 (circPTK2) inhibits TGF-β-induced epithelial-mesenchymal transition and metastasis by controlling TIF1γ in non-small cell lung cancer. Mol Cancer.

[82] Zhang M (2019). Downregulated circular RNA hsa_circ_0067301 regulates epithelial-mesenchymal transition in endometriosis via the miR-141/Notch signaling pathway. Biochem Biophys Res Commun.

[83] Meng J (2018). Twist1 regulates vimentin through Cul2 circular RNA to promote EMT in hepatocellular carcinoma. Cancer Res.

[84] Qu Y (2019). Circular RNA rno_circ_0004002 regulates cell proliferation, apoptosis, and epithelial-mesenchymal transition through targeting miR-342-5p and Wnt3a in anorectal malformations. J Cell Biochem.

[85] Zhang X (2019). Circular RNA circNRIP1 acts as a microRNA-149-5p sponge to promote gastric cancer progression via the AKT1/mTOR pathway. Mol Cancer.

[86] Li Y (2020). Circular RNA FOXO3 suppresses bladder cancer progression and metastasis by regulating MiR-9-5p/TGFBR2. Cancer Manag Res.

[87] Lu WY (2017). Roles of the circular RNA circ-Foxo3 in breast cancer progression. Cell Cycle.

[88] Xing Y (2020). Circular RNA circ-Foxo3 inhibits esophageal squamous cell cancer progression via the miR-23a/PTEN axis. J Cell Biochem.

[89] Zhang Y (2018). Identification of the tumor‑suppressive function of circular RNA FOXO3 in non‑small cell lung cancer through sponging miR‑155. Mol Med Rep.

[90] Feng XQ (2020). Circular RNA circHIPK3 serves as a prognostic marker to promote chronic myeloid leukemia progression. Neoplasma.

[91] Zheng Q (2016). Circular RNA profiling reveals an abundant circHIPK3 that regulates cell growth by sponging multiple miRNAs. Nat Commun.

[92] Chen X (2020). Circular RNA circHIPK3 modulates autophagy via MIR124-3p-STAT3-PRKAA/AMPKα signaling in STK11 mutant lung cancer. Autophagy.

[93] Bai H (2019). Exo-circRNAs: a new paradigm for anticancer therapy. Mol Cancer.

[94] Chen H (2018). Circ-ANAPC7 is upregulated in acute myeloid leukemia and appears to target the MiR-181 family. Cell Physiol Biochem.

[95] Li W (2017). Characterization of hsa_circ_0004277 as a new biomarker for acute myeloid leukemia via circular RNA profile and bioinformatics analysis. Int J Mol Sci.

[96] Hirsch S (2017). Circular RNAs of the nucleophosmin (NPM1) gene in acute myeloid leukemia. Haematologica.

[97] Shang J (2019). CircPAN3 mediates drug resistance in acute myeloid leukemia through the miR-153-5p/miR-183-5p-XIAP axis. Exp Hematol.

[98] Sun YM (2019). circMYBL2, a circRNA from MYBL2, regulates FLT3 translation by recruiting PTBP1 to promote FLT3-ITD AML progression. Blood.

[99] Ping L (2019). Silencing of circ_0009910 inhibits acute myeloid leukemia cell growth through increasing miR-20a-5p. Blood Cells Mol Dis.

[100] Yuan DM (2019). Identification of non-coding RNA regulatory networks in pediatric acute myeloid leukemia reveals circ-0004136 could promote cell proliferation by sponging miR-142. Eur Rev Med Pharmacol Sci.

[101] Han F (2020). hsa_circ_0001947 suppresses acute myeloid leukemia progression via targeting hsa-miR-329-5p/CREBRF axis. Epigenomics.

[102] Fan H (2018). Circular RNA-100290 promotes cell proliferation and inhibits apoptosis in acute myeloid leukemia cells via sponging miR-203. Biochem Biophys Res Commun.

[103] Wu DM (2018). Role of circular RNA DLEU2 in human acute myeloid leukemia. Mol Cell Biol.

[104] Chen LL (2020). The expanding regulatory mechanisms and cellular functions of circular RNAs. Nat Rev Mol Cell Biol.

[105] Chen JJ (2020). hsa_circ_0121582 inhibits leukemia growth by dampening Wnt/beta-catenin signaling. Clin Transl Oncol.

[106] Zhang L (2020). A novel circular RNA (hsa_circ_0000370) increases cell viability and inhibits apoptosis of FLT3-ITD-positive acute myeloid leukemia cells by regulating miR-1299 and S100A7A. Biomed Pharmacother.

[107] Papaioannou D (2020). Clinical and functional significance of circular RNAs in cytogenetically normal AML. Blood Adv.

[108] Wu W (2019). Downregulation of circ_0132266 in chronic lymphocytic leukemia promoted cell viability through miR-337-3p/PML axis. Aging (Albany NY).

[109] Xia L (2018). Circular RNA circ-CBFB promotes proliferation and inhibits apoptosis in chronic lymphocytic leukemia through regulating miR-607/FZD3/Wnt/β-catenin pathway. Biochem Biophys Res Commun.

[110] Wu Z (2020). Circ-RPL15: a plasma circular RNA as novel oncogenic driver to promote progression of chronic lymphocytic leukemia. Leukemia.

[111] Liu J (2018). Global identification of circular RNAs in chronic myeloid leukemia reveals hsa_circ_0080145 regulates cell proliferation by sponging miR-29b. Biochem Biophys Res Commun.

[112] Che H circ_0080145 enhances imatinib resistance of chronic myeloid leukemia by regulating miR-326/PPFIA1 axis. Cancer Biother Radiopharm.

[113] Ping L High circ_100053 predicts a poor outcome for chronic myeloid leukemia and is involved in imatinib resistance. Oncol Res.

[114] Gaffo E (2019). Circular RNA differential expression in blood cell populations and exploration of circRNA deregulation in pediatric acute lymphoblastic leukemia. Sci Rep.

[115] Hu J (2018). Circular RNA PVT1 expression and its roles in acute lymphoblastic leukemia. Epigenomics.

[116] Buratin A (2020). Large-scale circular RNA deregulation in T-ALL: unlocking unique ectopic expression of molecular subtypes. Blood Adv.

[117] Liu H (2019). Circ-SMARCA5 suppresses progression of multiple myeloma by targeting miR-767-5p. BMC Cancer.

[118] Song Y (2020). Hsa_Circ_0007841 enhances multiple myeloma chemotherapy resistance through upregulating ABCG2. Technol Cancer Res Treat.

[119] Zhou F (2020). Comprehensive profiling of circular RNA expressions reveals potential diagnostic and prognostic biomarkers in multiple myeloma. BMC Cancer.

[120] Feng Y (2019). CircRNA circ_0000190 inhibits the progression of multiple myeloma through modulating miR-767-5p/MAPK4 pathway. J Exp Clin Cancer Res.

[121] Chen F (2020). Circular RNA circ-CDYL sponges miR-1180 to elevate yes-associated protein in multiple myeloma. Exp Biol Med (Maywood).

[122] Hu Y (2019). A circular RNA from APC inhibits the proliferation of diffuse large B-cell lymphoma by inactivating Wnt/β-catenin signaling via interacting with TET1 and miR-888. Aging (Albany NY).

[123] Deng L (2019). Circ-LAMP1 promotes T-cell lymphoblastic lymphoma progression via acting as a ceRNA for miR-615-5p to regulate DDR2 expression. Gene.

[124] Guarnerio J (2016). Oncogenic role of fusion-circRNAs derived from cancer-associated chromosomal translocations. Cell.

[125] Tan S (2018). Circular RNA F-circEA produced from EML4-ALK fusion gene as a novel liquid biopsy biomarker for non-small cell lung cancer. Cell Res.

[126] Tan S (2018). Circular RNA F-circEA-2a derived from EML4-ALK fusion gene promotes cell migration and invasion in non-small cell lung cancer. Mol Cancer.

[127] Huang W (2019). circRNA circAF4 functions as an oncogene to regulate MLL-AF4 fusion protein expression and inhibit MLL leukemia progression. J Hematol Oncol.

[128] Dal Molin A (2019). CircRNAs are here to stay: a perspective on the MLL recombinome. Front Genet.

[129] Pan Y (2018). CircBA9.3 supports the survival of leukaemic cells by up- regulating c-ABL1 or BCR-ABL1 protein levels. Blood Cells Mol Dis.

[130] Ferreri AJ (2012). Anaplastic large cell lymphoma, ALK-positive. Crit Rev Oncol Hematol.

[131] Sibon D (2019). ALK-positive anaplastic large-cell lymphoma in adults: an individual patient data pooled analysis of 263 patients. Haematologica.

[132] Congras A (2018). Doxorubicin-induced loss of DNA topoisomerase II and DNMT1- dependent suppression of MiR-125b induces chemoresistance in ALK-positive cells. Oncotarget.

[133] Hoareau-Aveilla C (2019). miR-497 suppresses cycle progression through an axis involving CDK6 in ALK-positive cells. Haematologica.

[134] Hoareau-Aveilla C (2015). Reversal of microRNA-150 silencing disadvantages crizotinib-resistant NPM-ALK(+) cell growth. J Clin Invest.

