# Supplementary material for: From circRNAs to fusion circRNAs in hematological malignancies
Source: JCI Insight. 2021 Nov 8;6(21):e151513. doi: 10.1172/jci.insight.151513 (PMC8663548; doi:10.1172/jci.insight.151513)
Supplement: Supplemental data [file jciinsight-6-151513-s071.pdf]

Supplemental Table 1. CircRNAs involved in hematological diseases

Disease	circRNA	Host gene	Putative targets and mechanism	Expression	Phenotype	Clinical relevance	Refs	Databases
AML	circ-0004136	KCNQ5	miR-142	Up	Cell proliferation or growth	Potential therapeutic target	100	circad
	circ-0009910	MFN2	miR-20a-5p	Up	Cell proliferation	Potential therapeutic target	99	circBase
	circ-100290	SLC30A7	miR-203/Ras	Up	Cell proliferation, reduced apoptosis	Potential therapeutic target	102	circad
	circ-0004277	WDR37		Down	Decreased in refractory patients	Relapse biomarker	95	circad, circBase
	circ-0001947	AFF2	miR-399-5p/CREBRF	Down	Curbed cell proliferation	Restoration as treatment strategy	101, 102	circad, circBase
	circ-0121582	GSK3B	miR-224/SK3β	Down	Suppressed growth	Restoration as treatment strategy	105	circBase
	circANAPC7 circ-101141	ANAPC7	miR-181	Up	Promotes tumorigenic	Biomarker to monitor disease condition	94	cirbase, circad
	circ-0075001	NPM1			Correlates with NPM1 expression level	Potential biomarker for subgroup identification	96	circad, circBase
	circDLEU2 circ-0000488	DLEU2	miR-496/PRKACB	Up	Stimulates cell proliferation	Potential therapeutic target	103	circad, circBase
	circPAN3 hsa_circ_0100181	PAN3	miR153-5p, miR183-5p	Down	Decreases the antiapoptotic X-linked inhibitor of apoptosis protein expression and mediates doxorubicin resistance	Biomarker of resistance to doxorubicin	97	circad, circBase
	circMYBL2	MYBL2	RBP PTBP1	Up in patients with FLT3-ITD mutations	Impairs proliferation and overcomes acquired resistance to FLT3 kinase	Biomarker for FLT3-IDT–mutated patients, potential therapeutic target	98	circBase
	circ-0000370	FLI1	miR-1299/S100A7A	Abnormally expressed in FLT3-ITD–positive cells	Promotes viability and suppresses apoptosis	Potential therapeutic target for FLT3-IDT–positive patients	106	circBase
	circFBXW7	FBXW7		Down	Tumor suppressor	Restoration as treatment strategy	107	NA
CLL	circ-0132266	MTO1	miR-337-3p/PML	Down	Influence on cell viability	Biomarker, restoration as treatment strategy	108	circBase
	circ-CBFB circ-0000707	CBFB	miR-607/FZD3/Wnt/β-catenin	Up	Promotes proliferation and inhibits apoptosis	Potential therapeutic target	109	circad, circBase
	circRPL15 circ-0064574	RPL15	miR-146b-3p/RAF1 axis	Up	Increases cell viability	Prognosis biomarker (detectable in plasma)	110	circBase
CML	circHIPK3 circ-0000284	HIPK3	miR-124/B4GAL1/NF-κB	Up	Promotes cell proliferation	Prognosis biomarker (detectable in PBMCs and serum)	90	circBase
	hsa_circ_0080145	TNS3	miR-29b/ABL1, BCR-ABL, miR-326/PPFIA1 axis	Up	Protumor, enhances imatinib resistance	Resistance biomarker	111, 112	circad, circBase
	circ-100053	MFN2		Up in resistance to imatinib		Resistance biomarker	113	
ALL	circPVT1 circ-0001821	PVT1	miR-let-7, miR-125/c-Myc, Bcl-2	Up	Induces cell proliferation, inhibits apoptosis	Potential therapeutic target	115	circad, circBase
	circZNF609	ZNF609	miR-181a-5p, miR-125a-5p (putative targets)	Up in immature T-ALL	Promotes T cell proliferation	Potential therapeutic target	46	NA
	circAF4	AF4	miR-128-3p/MLL-AF4	Up	Promotes leukemogenesis in vitro and in vivo	Potential therapeutic target	127	NA
	circPAX5	PAX5	miR-124 (putative target)	Up			114	NA
DLBCL	circAPC circ-0127621	APC	miR-888/APC, TET1/APC, inhibits Wnt/β-catenin	Down	Inhibits proliferation	Circulating biomarker	122	circBase
	circIKZF3	IKZF3		Up			62	NA
MCL	circCDYL	CDYL	hsa-miR-129-5p, hsa-miR-3163, hsa-miR-4662a-5p, hsa-miR-101-3p, and hsa-miR-186-5 (potential targets)	Up	Promotes cell proliferation	Diagnosis biomarker	121	
T-LBL	circLAMP1 circ-101303	LAMP1	miR-615-5p/DDR2	Up	Promotes cell proliferation, inhibits apoptosis	Potential therapeutic target	123	NA
MM	circSMARCA5	SMARCA5	miR-767-5p	Down	Inhibits proliferation and promotes apoptosis	Restoration as treatment strategy	117	NA
	circPTK2 circ-0005273	PTK2	miR-1298-5p	Up	Promotes tumor progression	Potential therapeutic target	119	circad, circBase
	circAFF2	AFF2	miR-638	Down	Tumor suppressor	Diagnosis and prognosis biomarker	119	circad, circBase
	hsa_circ_0007841	SEC61A1	ABCG2	Up	Induces chemotherapy resistance	Biomarker for drug resistance	118	circBase
	hsa_circ_0000190	CNIH4	miR-767-5p/MAPK4	Down	Inhibits cell viability and proliferation and induces apoptosis	Restoration as treatment strategy	120	circad, circBase
	circCDYL	CDYL	miR-1180/YAP	Up	Facilitates MM progression	Biomarker, potential therapeutic target	121	NA

AML, acute myeloid leukemia; CLL, chronic lymphocytic leukemia; CML, chronic myeloid leukemia; ALL, acute lymphoblastic leukemia; DLBCL, diffuse large B cell lymphoma; MCL, mantle cell lymphoma; T-LBL, T cell lymphoblastic lymphoma; MM, multiple myeloma.
